# Two new South American species of *Solanum* section *Crinitum* (Solanaceae)

**DOI:** 10.3897/phytokeys.1.661

**Published:** 2010-11-01

**Authors:** Frank T. Farruggia, Lynn Bohs

**Affiliations:** Department of Biology, 257 S. 1400 E. Salt Lake City, Utah 84112, U.S.A.

**Keywords:** Andes, Brazil, cerrado, Ecuador, new species, Peru, *Solanum*, *Solanum falciforme*, *Solanum pseudosycophanta*

## Abstract

Two new species of Solanum section Crinitum are described here. Solanum falciforme Farruggia, **sp. nov.**, closely resembles Solanum crinitum and Solanum lycocarpum, but differs by the presence of falcate trichomes on the young growth. It is endemic to the cerrado and adjacent woodlands of Distrito Federal, Bahia, Goiás and Minas Gerais, Brazil. The other species, Solanum pseudosycophanta Farruggia, **sp.nov.**, has close affinities to Solanum sycophanta butdiffers from the latter in having prominent long-stalked stellate hairs along the stem, calyx, petiole and the adaxial surface of the leaf, in contrast to Solanum sycophanta which is glabrous or pubescent with sessile to short-stalked multangulate hairs. This species is narrowly distributed in tropical montane forests of northern Peru and southern Ecuador.

## Abstract (sp)

Se describen aquí dos especies nuevas de Solanum sección Crinitum. Solanum falciformeFarruggia, **sp.nov.**, se asemeja a Solanum crinitum y Solanum lycocarpum, pero difiere de las otras dos por la presencia de tricomas falcadas en los partes jóvenes de la planta. La nueva especie es endémica al cerrado y bosques adyacentes de Distrito Federal, Bahía, Goiás y Minas Gerais, Brasil. La otra especie, Solanum pseudosycophanta Farruggia, **sp.nov.,** tiene estrechas afinidades con Solanum sycophanta pero difiere de ésta por tener pelos estrellados con tallos largos en las ramas, cálices, pecíolos y en la superficie adaxial de la hoja; al contraste, Solanum sycophanta está glabra o pubescente con pelos multangulados sesiles o con tallos cortos. Esta especie está estrechamente distribuida en los bosques tropicales montanos del norte de Perú y sur del Ecuador.

## Introduction

The economically important genus Solanum L., which includes tomato (Solanum lycopersicum L.), potato (Solanum tuberosum L.), and eggplant (Solanum melongena L.), is currently the focus of an initiative to provide online descriptions and taxonomic information for all Solanum species (the PBI Solanum project; www.solanaceaesource.org). Detailed taxonomic study of Solanum section Crinitum Whalen ex A. Child has revealed undescribed species within the section, two of which are treated below.

Solanum section Crinitum is comprised of large trees and shrubs, 2–30 m tall, with large, 3 to 8.5 cm diameter, purple flowers fading to white in some taxa. The fruits are some of the largest known in the genus, ranging in size from 1 cm up to 15 cm in diameter, and have a swollen to knobby calyx at maturity. The group as circumscribed by Whalen (1984) and Nee (1999) includes about 14 species. One of these, Solanum mitlense Dunal, is endemic to Mexico, while the rest are restricted to tropical South America, with highest diversity along the eastern slopes of the Andes from Colombia to Bolivia.

## Taxonomic treatments

### Solanum falciforme

Farruggia sp. nov.

urn:lsid:ipni.org:names:77107761-1

Figs. 1 2 

#### Latin

*Frutex vel arbuscula, 1–3 (–4) m × ca. 2–5 cm diametro, flores magnae, corollis 3.5–4.5 cm diametro, rotato-stellatis, fructus globosus, puberulus vel glabrisculus, 5–7.5 cm diametro.* Solano crinito *et* S. lycocarpo *affinis sed pilis falcatis longistipitatis stellatis caulium differt.*

#### Type.

 **Brazil:** Distrito Federal: Brasília, on road to Gama, DF16, 3–7 km from junction with BR040, 15°58S, 48°02W , 1100 m, 10 Jul 1984, S.A. Mori et al. 16658 (holotype: NY!; isotype: MO-3580598!).

#### Description.

 Shrub or small tree 1–3 (–4) m × ca. 2–5 cm dbh. Trunk with sharp, stout broad-based prickles, the bark grey-brown to reddish-dark brown, smooth to slightly roughened; flowering stems armed with broad-based prickles, very densely pubescent with sessile to short-stalked light tan multangulate-stellate hairs, the apex 0.1–0.3 mm in diameter, the rays 7–10+, moderately to densely pubescent with falcate long-stalked stellate hairs, the stalks ca. 4–6.2 mm, multiseriate, the apex 0.1–0.3 mm in diameter, the rays 5–7. Sympodial units difoliate, geminate. Leaves simple, the blades ca. 19–25 × 7–17 cm or more, ca. 2.5 times as long as wide, lanceolate, coriaceous, slightly discolorous, the fresh and dried leaves light green adaxially, lighter green abaxially, the adaxial surface very densely pubescent when young with stalked stellate hairs, these nearly absent on older plants, the stalks ca. 0.1–0.3 mm, multiseriate at the base, the rays 7–8, the midpoints ca. 0.1 mm, these mixed with abundant short simple glandular hairs beneath the stellate pubescence, the abaxial surface very densely pubescent with golden-tan multiseriate-stalked porrect-stellate hairs, the stalks 0.2–0.4 mm, the rays 7–10, the midpoints absent; major veins 5–6 on either side of midvein, abundantly armed with broad-based prickles and falcate long-stalked stellate hairs; base cordate to oblique; margin entire to deeply repand; apex acute to obtuse; petioles (1–) 3–5 cm, densely pubescent with hairs like those of the young stems. Inflorescences 3–9.5 cm, extraaxillary, unbranched, with 8–15 flowers, the plants strongly andromonoecious, with one to few hermaphroditic flower(s) at the base of the inflorescence and all other flowers functionally staminate, the axes densely stellate-pubescent with hairs like those of the stems, armed or unarmed; peduncle 18–22 mm; rachis 2–8 cm; pedicels 4–10 mm in flower and fruit, densely congested, spaced 1–4 mm apart, articulated at base. Flowers 5-merous. Calyx ca. 2.5 cm long, the tube at anthesis 2–3 mm, the lobes ca. 20 × 2 mm, the apex acute, the abaxial surface densely pubescent with short-stalked to sessile porrect-stellate hairs and falcate long-stalked stellate hairs, armed or unarmed; fruiting calyx tube becoming slightly thickened and accrescent with maturity, the lobes 7–15 × 3–8 mm, slightly reflexed, subtending but not enclosing the fruit. Corolla 3.5–4.5 cm in diameter, 16–23 mm long, stellate to rotate-stellate with abundant interpetalar tissue, lobed for more than half of its length, membranaceous, violet to blue, the tube 6–8.2 mm, the lobes 16–19 × 3.5–4 mm, deltate, moderately pubescent adaxially with sessile to short-stalked multangulate or porrect-stellate hairs, the rays 5–10, the midpoints often pronounced, ca. 0.1–0.2 mm long, densely pubescent abaxially with sessile to short-stalked porrect-stellate and falcate long-stalked stellate hairs. Stamens equal, the filament tube 0–0.1 mm, the free part of the filaments 1.5–1.8 mm, glabrous; anthers ca. 13 × 2.8 mm, tapered, connivent, yellow, the pores directed distally, opening into longitudinal slits with age, the connective stellate-pubescent. Ovary densely pubescent with sessile stellate hairs; style in hermaphroditic flowers 14–15 × 0.2–0.5 mm, cylindrical, curved at apex, glabrous or sparsely pubescent in lower half with sessile stellate or short-stalked unbranched glandular hairs; style in staminate flowers vestigial; stigma capitate, slightly bilobed. Fruit a berry, 5–7.5 cm in diameter, globose, likely green at maturity, powdery pubescent with stellate hairs. Seeds unknown.

#### Distribution.

Endemic to Brazil. Found in cerrado and along roadsides, 380–1300 m in elevation, common in States of Goiás and Distrito Federal, but also occurring in Bahia and Minas Gerais.

#### Ecology.

Flowering specimens were collected in January–December. Fruiting specimens were collected in January, March, June and July.

#### Conservation status.

 According to the IUCN Red List Categories (IUCN 2010), Solanum falciforme is classified asVU-B1a+biii; A2c (Vulnerable). Populations of this species are located near expanding population centers leading to highly fragmented populations. The extent of occupancy is estimated to be less than 20,000 km2. There is also a continuing decline in suitable habitat in these regions due to deforestation and the establishment of new settlements.

#### Local names.

 Brazil: Lobeiro (Costich 1017); fruto do lobo (Macedo 3245, Heringer 10718).

**Figure:**
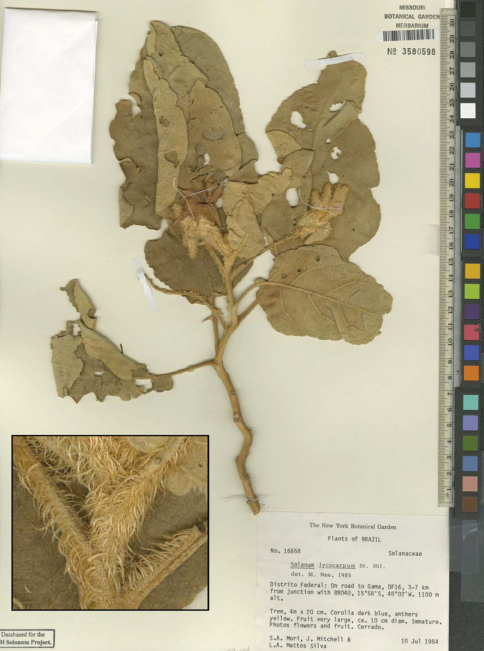
**Figure 1.** Solanum falciforme Farruggia.Image of Isotype [S.A. Mori et al. 16658 (MO)]. Detail of falcate hairs (inset).

**Figure:**
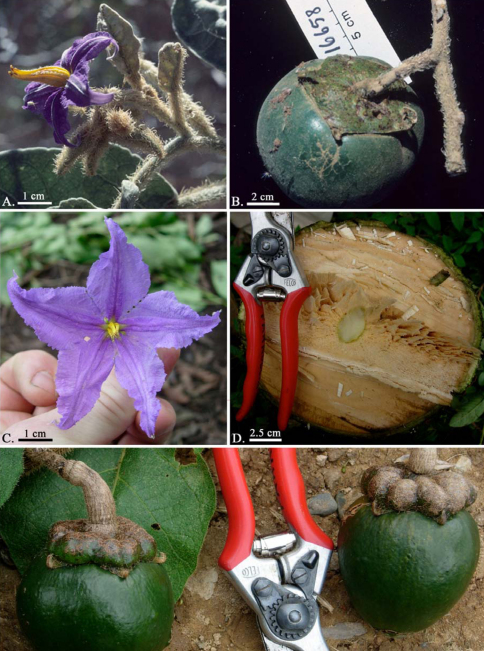
**Figure 2.** **A-B** Solanum falciforme Farruggia. Images of type collection [S. Mori et al. 16658]. **A** Staminate flower with pubescent anthers and falcate hairs on axes **B** Mature fruit showing powdery pubescence and expanded calyx tube. Photos by S. Mori. **C-E** Solanum pseudosycophanta Farruggia [L. Bohs et al. 3784]. **C** Staminate flower **D** Cross-section of trunk showing abundant secondary xylem and spongy pith **E** Mature fruits showing knobby calyx with thickened lobes reduced to points. Photos by F.T. Farruggia.

#### Discussion.

 Within Solanum section Crinitum, Solanum falciforme most closely resembles Solanum lycocarpum A. St.-Hil., Solanum gomphodes Dunal and Solanum crinitum Lam. All four species have pubescence of sessile to short-stalked porrect-stellate hairs and distributions centered in eastern Brazil. Solanum gomphodes can be easily distinguished from Solanum falciforme by its sessile leaves (vs. petioles usually 3–5 cm long in Solanum falciforme) and exclusively short-stalked stellate hairs. Solanum lycocarpum and Solanum crinitum are sympatric with Solanum falciforme, but the former two species have much broader distributions throughout South America. Solanum falciforme is easily distinguished from S. lycocarpum and S. crinitum by the presence of macroscopic falcate (sickle-shaped) long-stalked bristly hairs on the young stems as well as the inflorescence and calyx. Collections of Solanum crinitum often have similar long-stalked trichomes on the young stems, inflorescence and calyx; however the stalks of these hairs are straight. Solanum lycocarpum is similar to the other three taxa in its abundant pubescence of short-stalked stellate hairs, but the distinctive long-stalked hairs found in Solanum falciforme are noticeably absent.

#### Etymology.

The name is derived from the Latin “falcatus” describing the sickle-shaped long hairs characteristic of this species.

#### Representative specimens.

 **Brazil:** sin loc,1821, L. Riedel 3013 (MO, NY, US). Bahia: Barreiras, estrada para o Aeroporto de Barreiras, entre 5 a 15 km a partir da sede do município, 11 Jun 1992, A.M.V. de Carvalho et al. 4028 (NY). Distrito Federal: Chapada da Contagem, Vegetação de Transição, 6 Oct 1981, A.L.V. Atta 6 (F); estrada que vai de Brasília ao Gama, próximo ao balão que vai para Taguatinga, 11 Nov 1981, R. Batista 10 (F); Rodovia Brasília-Uruaçu, 15 Dec 1964, R.P. Belém & J.M. Mendes 31 (F, US); região da Palma, beira da estrada, 1980, V. de Carvallho dos Anjos 15 (F); Chapada de Contagem, 15°37'S, 47°58'W , 6 Oct 1981, E.A. Costa 10 (F); Parque Nacional, 8 Nov 1981, D. Costich 1017 (F); Brasília, Planaltina, EMBRAPA-CPAC Reserve, 1000 m, 22 Jul 1982, D. Costich 1091 (F, NY); Brasília, Planaltina, EMBRAPA-CPAC Reserve, 1000 m, 23 Jul 1982, D. Costich 1092 (F, NY); Região da Palma, campo sujo, 15°34'S, 48°02'W , 1300 m, 3 Jun 1981, R.S. Ganem & C.M.S. Mello 20 (F); Brasília, no Centro Olímpico da UnB, 2 Jun 1980, A.E. Heringer Salles et al. 164 (NY); Brasília, 9 Oct 1979, E.P. Heringer et al. 2288 (MO, NY); Brasília, proximidades de Santo Antônio do Descoberto – Goiás, 25 Oct 1979, E.P. Heringer et al. 2607 (MO); Brasília, bacia do Rio São Bartolomeu, 10 Apr 1980, E.P. Heringer et al. 4301 (NY, US); Brasília, Cabeça do Viado, 3 Mar 1961, E.P. Heringer 8060 (US); Brasília, Sobradinho, 8 Jan 1965, E.P Heringer 10180 (NY); Brasília, 21 May 1978, E.P. Heringer 16747 (F, MO); Brasília, João Pinheiro, 20 Aug 1981, E.P. Heringer 18078 (F, US); Brasília, ca. 25 km S. of Brasília on road to Belo Horizonte, 700 m, 26 Aug 1964, H.S. Irwin & T.R. Soderstrom 5610 (F, NY, US); CAESB, 4.4 km NNE do centro de Brasília, G.S. Koury 23 (F); CAESB, 4.4 km NNE do centro de Brasília, 30 May 1979, S. Kunzler 23 (F); Corrégo Capão da Erva, 10 Nov 1981, M.F. Luz 13 (F); vicinity of Universidade de Brasília, 29 Jul 1965, R.T. Martin 486 (GH); região da Palma, 15°34'S, 48°02'W , 9 Jun 1981, T.L.F. Martins 18 (F); região da Palma, 15°34'S, 48°02'W , 9 Jun 1981, M.L.P. Matricuela 17 (F); região da Palma, 15°34'S, 48°02'W , 1200 m, 9 Jun 1981, R. Matrícula de Oliveira L. 11 (F); Brasília, campo sujo, próximo á estrada, 15°34'S, 48°02'W , 1000 m, C.M.S. Mello & R.S. Ganem 24 (F); Córrego Capão da Mata, 15°45'S, 47°43'W , 1000 m, 10 Nov 1981, A.G. Miranda 20 (F); região da Palma, 15°34'S, 48°02'W , 9 Jun 1981, A.G. Moreira 15 (F); Brasília, University Campus, along road parallel to Lago Paranoa, 6 Oct 1975, F.H. Oldenburger & V.V. Mecenas 1698 (NY); campos campestres, Córrego Capão da Erva, 15°45'S, 47°43'W , 1000 m, 10 Nov 1981, M.A.F. de Oliveira 20 (F); Brasília, 13 Nov 1958, Edm. Pereira 4612 (US); Brasília, Fazenda Agua Limpa, University of Brasília field station, near Vargem Bonita, c. 18 km. SSW of Brasília TV tower, 9 Jun 1976, J.A. Ratter et al. R3134 (NY); Brasília, Entrada da Fazenda Sucupira – CPAC, 1100 m, 17 Nov 1987, L.A. Skorupa & W.L. Werneck 53 (MO, NY); Brasília, campus of the Universidade de Brasília, 1050 m, May 1973, Taxonomy class of the University of Brasília 129 (MO, US); Brasília, Chapada da Contagem, 6 Oct 1981, M.P. Valle & M.L. Batista 6 (F, NY); Brasília, próximo ao setor de clubes, 1100 m, 19 Apr 1982, E.M.X. Vieira 1 (NY); Brasília, próximo ao lago Paranoá, UnB. Centro Olímpico, 1100 m, 26 Jun 1982, E.M.X. Vieira 20 (F); Brasília, Fazenda Chapadinhoa em Jose Pires, Apa da Cafuringa, 1000 m, 25 Sep 1990, R.F. Vieira et al. 517 (NY). Goiás: cerrado and rocky hillside ca. 25 km by road SW of Monte Alegre de Goiás, northern spur of Serra Atalaia, 600 m, 13 Mar 1973, W.R. Anderson 6999 (F, MO, NY, US); Chapada dos Veadeiros, 13 km by road S of Terezina, 1000 m, 19 Mar 1973, W.R. Anderson 7501 (F, MO, NY, US); Serra do Caiapó, ca. 16 km (straight line) S of Caiapônia, 800 m, 1 May 1973, W.R. Anderson 9549 (F, MO, NY, US); Verrado do Loteamento Santa Maria próximo a quadra 19, 23 May 1981, A.C.S. Berçot 35 (F); Hidrolândia, Morro Feio, 5 km N Hidrolandia, 950 m, 7 Apr 1988, R.R. Brooks et al. BRASPEX #1 (NY); Niquelândia, nas margens do Rio Tocantinzinho, 450 m, 22 Jul 1995, T.B. Cavalcanti et al. 1603 (NY); region of the Chapada dos Veadeiros, 20 km N of Sao Joao da Alianca, 14°30'S, 47°30'W , 16 Apr 1956, E.Y. Dawson 14283 (US); Município de Jataí, 10 km north of Jataí, ca. 900 m, 15 Oct 1968, G. Eiten & L.T. Eiten 9331 (NY); Alto Paraíso de Goiás, Fazenda Santo Antônio, 15 Nov 1997, J.M. Felfili 371 (NY); Planaltina, Rod. GO-118, 45 km S de São Gabriel de Goiás, 8 May 2000, G. Hatschbach et al. 70610 (NY); Santo Antônio do Descoberto, 26 Feb 1980, E.P. Heringer et al. 3492 (NY); Santo Antônio do Descoberto, 26 Feb 1980, E.P. Heringer et al. 3494 (NY, US); Formosa, 20 Oct 1966, E.P. Heringer 10718 (US); Formosa, 16 Oct 1965, E.P. Heringer 10720 (NY); Cristalina, ca. 6 km S of Cristalina, Serra dos Cristais, 1175 m, 3 Nov 1965, H.S. Irwin et al. 9892 (F, NY, US); Cristalina, ca. 5 km S of Cristalina, Serra dos Cristais, 1200 m, 2 Mar 1966, H.S. Irwin et al. 13285 (F, NY, US); 75 km N of Corumbá de Goiás on road to Niquelândia, Goiás in valley of Rio Maranhão, 700 m, 22 Jan 1968, H.S. Irwin et al. 18982 (GH, F, US); Alto Paraíso de Goiás, ca. 10 km S of Alto do Paraíso, 1000 m, 22 Mar 1969, H.S. Irwin et al. 24919 (F, NY); Alto Paraíso de Goiás, ca. 20 km N of Alto do Paraíso, 1250 m, 23 Mar 1971, H.S. Irwin et al. 33066 (NY); Niquelândia, ca. 8 km S of Niquelândia, 23 Jan 1972, H.S. Irwin et al. 34878 (F, NY); Goiânia, 3 Jul 1951, A. Macedo 3245 (MO, US); Serra dos Pirineus, 75 km N of Corumbá de Goiás on road to Niquelândia, in valley of Rio Maranhão, 700 m, 22 Jan 1968, H. Maxwell 18982 (MO, NY); Niquelândia, margem direita do Rio Bagagem, próximo a Barra do Baga Gem/Tocantins (Maranhão), região da Serra Negra, 380 m, 26 Jul 1995, B. Walter et al. 2521 (MO). Minas Gerais: Parque Nacional Grande Sertão Veredas, 30 Apr 1999, R. Rodrigues-da Silva et al. 281 (NY).

### Solanum pseudosycophanta

Farruggia sp. nov.

urn:lsid:ipni.org:names:77107762-1

Figs. 2 3 

#### Latin

*Arbor, (5–) 10–20 (–30) m × 7–40 cm diametro, truncus aculeis crassis armatus, flores magnae, corollis 5–8.5 cm diametro, stellatis ad rotato-stellatis, fructus ellipticus, glaber, 6–10 cm diametro.* Solano sycophantae *affinis sed pilis longistipitatis stellatis superficialum foliorum caulium et calycium differt.*

#### Type.

 **Peru:** Cajamarca: Provincia San Ignacio, road to El Chaupe north of San Ignacio, 3 km north of town of Marizaua, 5°08'57"S, 79°01'36"W , 1591 m, 17 Dec 2007, S. Stern et al. 178 (holotype: USM!; isotypes: NY!, UT!).

#### Description.

Tree (5–) 10–20 (–30) m × 7–40 cm dbh. Trunk with sharp, stout broad-based prickles, the bark light brown to reddish, thin with shallow fissures; flowering stems often unarmed, nearly glabrous to densely pubescent with stalked reddish-tan multangulate-stellate hairs, the apex 0.7–0.9 mm in diameter, the rays 8–10. Sympodial units difoliate, geminate. Leaves simple, the blades 10–40 (–50) × 7–25 (–30) cm or more, length to width ratio ca. 1.5–1.7:1, elliptical, ovate to lanceolate, chartaceous, discolorous, the fresh and dried leaves dark green adaxially, light green to whitish- or yellowish-green abaxially, the adaxial surface moderately pubescent with simple glandular hairs and stalked porrect-stellate hairs, the stalks 0.5–3 (–5) mm, multiseriate, the rays 4–8, the midpoints 0.1–0.3 mm, eglandular, the abaxial surface densely pubescent with whitish-golden stalked porrect-stellate hairs, the stalks 1–1.5 mm, the rays 4–6, the midpoints ca. 0.5 mm; major veins 6–8 on either side of midvein, unarmed; base cordate to oblique; margin entire to shallowly lobed, the apex of lobes broadly rounded to acute; apex acute to acuminate; petioles 5–6 (–9) cm, densely pubescent with hairs like those of the young stems. Inflorescences 2–10 cm, extra-axillary, branched, with 10+ flowers, the plants strongly andromonoecious, with one to few hermaphroditic flower(s) near the base of the inflorescence and all other flowers functionally staminate, the axes densely stellate-pubescent with hairs like those of the stems, unarmed; peduncle 10–55 mm; rachis 2.5–8 cm; pedicels 8–25 mm in flower and fruit, densely congested, spaced 1–2 (–5) mm apart, articulated at base. Flowers 5-merous. Calyx 10–20 mm long, the tube at anthesis 2–3 mm, the lobes ca. 11 × 4 mm, the apex acute to acuminate, the abaxial surface densely stellate-pubescent with hairs like those of the stems, unarmed; fruiting calyx becoming inflated, knobby and woody, the lobes remaining as thick points, subtending the fruit. Corolla 5–8.5 cm in diameter, stellate to rotate-stellate with moderate interpetalar tissue, lobed for more than half of its length, membranaceous, light purple to violet, the tube 12–20 mm, the lobes 20–35 × 3.5–5 mm, lanceolate, sparsely pubescent adaxially with sessile multangulate stellate hairs, the rays 1–10, densely pubescent abaxially along central portion of lobes with sessile porrect-stellate hairs. Stamens slightly unequal, the filament tube ca. 2.2 mm, the free part of the filaments ca. 2 mm, glabrous; anthers 11–16 × ca. 1.5 mm, tapered, connivent, yellow, the pores directed distally, opening into longitudinal slits at maturity. Ovary slightly pubescent with simple glandular and porrect-stellate hairs, becoming glabrous with age; style in hermaphroditic flowers 16–18 × ca. 1.1 mm, cylindrical, strongly curved at apex, slightly to moderately pubescent at base with hairs similar to those of the ovary; style in staminate flowers 4–4.4 × ca. 0.2 mm, cylindrical, straight at apex, slightly to moderately pubescent at base with hairs similar to those of the ovary; stigma capitate, slightly bilobed. Fruit a berry, 6–10 cm in diameter, ellipsoidal to turbinate, green and juicy at maturity, glabrous, the pericarp thick with sclerified inclusions. Seeds 4–6.5 × 3.5–4 mm, strongly flattened, reniform, reddish brown to light brown, rugose.

**Figure:**
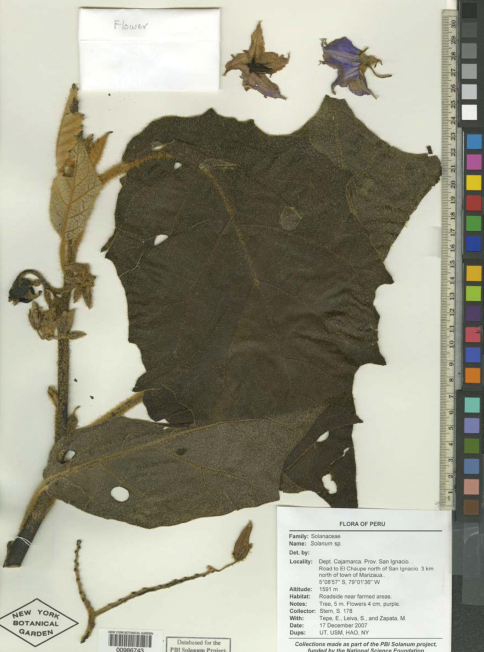
**Figure 3.** Solanum pseudosycophanta Farruggia.Image of isotype[S. Stern et al. 178 (NY)].

#### Distribution.

 Restricted to northern Peru and southern Ecuador in clearings and open places in disturbed, transitional and montane tropical forest, 900–1900 m in elevation.

#### Ecology.

Flowering specimens were collected in May, and October-December. Fruiting specimens were collected in May, and November-December.

#### Conservation status.

 According to the IUCN Red List Categories (IUCN 2010), Solanum pseudosycophanta is classified asVU-B1a+biii; A2c; D1 (Vulnerable). Populations of this species are located near expanding population centers leading to highly fragmented populations. The extent of occupancy is estimated to be less than 20,000 km2, less than 10 locations, and there are estimated to be less than 1,000 mature individuals across its range. There is also a continuing decline in suitable habitat in these regions due to deforestation and the establishment of new settlements.

#### Local names.

 Peru: Lucuma de oso (Bohs et al. 3784).

#### Uses.

 Used for firewood (Bohs et al. 3784).

#### Discussion.

 Within Solanum section Crinitum, Solanum pseudosycophanta most closely resembles Solanum sycophanta Dunal. The distribution of these taxa overlaps in Peru and Ecuador; however, Solanum sycophanta is more widespread throughout the Andes, while Solanum pseudosycophanta is restricted to northern Peru and southern Ecuador. These two species have large (4–8 cm) elliptical to round, glabrous fruits, more or less spiny trunks, and predominantly entire leaves at maturity. Solanum pseudosycophanta differs from Solanum sycophanta in having prominent long-stalked stellate hairs on the stem, inflorescence, and adaxial surface of the leaf. In Solanum sycophanta the stems and petioles lack long-stalked stellate hairs, and are glabrous or pubescent with sessile to short-stalked multangulate hairs. The calyx also differs between these two taxa; Solanum pseudosycophanta has calyx lobes with acute apices that cover most of the corolla in bud, while Solanum sycophanta has a calyx with shorter truncate calyx lobes that only partially cover the petals in bud. Furthermore, Solanum pseudosycophanta in fruit has a knobby calyx with thick pointed lobes, whereas Solanum sycophanta has a smaller rounded calyx without the pointed lobes.

#### Etymology.

The name Solanum pseudosycophanta was chosen because of the similarity of this taxon to Solanum sycophanta.

#### Representative specimens.

 **Ecuador:** Zamora Chinchipe: road between El Progreso and Guaramizal, ca. 3 km after turnoff from Vilcabamba-Zumba road at El Progreso, 4°48'23"S, 79°07'26"W , 1430 m, 28 Mar 2005, L. Bohs et al. 3322 (NY, UT); Palanda, región de la Cordillera del Cóndor, parroquia San Francisco de Vergel, riberas del Río Vergel, entre Santa Rosa y La Canela, 04°49'07"S, 79°01'41"W , 1200 m, 6 Mar 2007, W. Quizhpe & A. Wisum 2491 (MO, NY). **Peru:** Amazonas: Bongará, Shillac, N by trail from Pedro Ruíz, 5°49'S, 78°01'W , 2300 m, 31 Aug – 2 Sep 1983, D.N. Smith & S. Vasquez S. 4890 (MO, NY); Bongará, road from Pedro Ruiz to Moyobamba, 15 km east of Pedro Ruiz just before town of Carrera, 5°52'35"S, 77°55'45"W , 1780 m, 14 Dec 2007, S. Stern et al. 137 (NY, USM, UT); Bongará, Shillac, 1900 m, 8 May 1991, K. Young & M. Eisenberg 417 (MO, NY). Cajamarca: San Ignacio, San Ignacio, El Chaupe, 5°10'50.1"S, 79°03'25.0"W , 1800 m, 10 Oct 2010, F.T. Farruggia et al. 2711 (HAO, MO, NY, PLAT, USM, UT); San Ignacio, San Ignacio, La Mora, 5°05'S, 79°03'W , 1800 m, 6 Feb 1996, J. Campos & O. Díaz 2450 (USM); San Ignacio, Santuario Nacional Tabaconas-Namballe, pampa Limón, zona de amortiguamiento, 5°17'29"S, 79°16'32"W , 1980 m, 23 Nov 1998, C. Diaz et al. 10125 (MO, NY, USM); San Ignacio, San José de Lourdes, Santo Tomás, NE del Marañón RENOM, 04°55'S, 78°50'W , 1950 m, 1 Nov 1995, V. Quipuscoa S. 418 (MO, NY); San Ignacio, Dist. San José de Lourdes, Villa Rica, 4°55'S, 78°50'W , 1750 m, 27 Oct 1995, R. Vasquez et al. 20426 (MO, NY); San Ignacio, San José de Lourdes, Villa Rica, 5°03'42"S, 78°53'32"W , 1550 m, 27 May 2010, L. Bohs et al. 3784 (UT); San Ignacio, San José de Lourdes, Villa Rica, 5°03'39.8"S, 78°53'26.1"W , 1625 m, 11 Oct 2010, F.T. Farruggia et al. 2736 (HAO, MO, NY, PLAT, USM, UT); San Ignacio, San José de Lourdes, Buenos Aires del Parco, 5°04'07"S, 78°32'35"W , 1700 m, 16 Jul 2001, E. Vicuña et al. 465 (USM).

## Supplementary Material

XML Treatment for Solanum falciforme

XML Treatment for Solanum pseudosycophanta
